# Parent Priorities in End-of-Life Care for Children With Cancer

**DOI:** 10.1001/jamanetworkopen.2023.13503

**Published:** 2023-05-01

**Authors:** Prasanna Ananth, Meghan Lindsay, Sophia Mun, Sarah McCollum, Veronika Shabanova, Sophia de Oliveira, Sarah Pitafi, Rebecca Kirch, Xiaomei Ma, Cary P. Gross, Jackelyn Y. Boyden, Chris Feudtner, Joanne Wolfe

**Affiliations:** Department of Pediatrics, Yale School of Medicine, New Haven, Connecticut; Yale Cancer Outcomes, Public Policy and Effectiveness Research Center, New Haven, Connecticut; Yale Cancer Outcomes, Public Policy and Effectiveness Research Center, New Haven, Connecticut; Kaiser Permanente Washington Health Research Institute, Seattle; Department of Pediatrics, Yale School of Medicine, New Haven, Connecticut; Department of Pediatrics, Yale School of Medicine, New Haven, Connecticut; Yale University, New Haven, Connecticut; University College London, London, United Kingdom; National Patient Advocate Foundation, Washington, District of Columbia; Yale Cancer Outcomes, Public Policy and Effectiveness Research Center, New Haven, Connecticut; Department of Chronic Disease Epidemiology, Yale School of Public Health, New Haven, Connecticut; Yale Cancer Outcomes, Public Policy and Effectiveness Research Center, New Haven, Connecticut; Department of Internal Medicine, Yale School of Medicine, New Haven, Connecticut; Department of Family and Community Health, University of Pennsylvania School of Nursing, Philadelphia; Justin Michael Ingerman Center for Palliative Care, Children’s Hospital of Philadelphia, Philadelphia, Pennsylvania; Justin Michael Ingerman Center for Palliative Care, Children’s Hospital of Philadelphia, Philadelphia, Pennsylvania; Department of Pediatrics, Medical Ethics, and Health Policy, Perelman School of Medicine, University of Pennsylvania, Philadelphia; Department of Pediatrics, Massachusetts General Hospital, Harvard Medical School, Boston

## Abstract

**IMPORTANCE:**

Robust quality measures to benchmark end-of-life care for children with cancer do not currently exist; 28 candidate patient-centered quality measures were previously developed.

**OBJECTIVE:**

To prioritize quality measures among parents who lost a child to cancer.

**DESIGN, SETTING, AND PARTICIPANTS:**

This survey study was conducted using an electronic, cross-sectional discrete choice experiment (DCE) with maximum difference scaling from January to June 2021 in the US. In each of 21 questions in the DCE, participants were presented with a set of 4 quality measures and were asked to select the most and least important measures within each set. All 28 quality measures were presented an equal number of times in different permutations. In the volunteer sample, 69 eligible bereaved parents enrolled in the study; 61 parents completed the DCE (participation rate, 88.4%).

**MAIN OUTCOMES AND MEASURES:**

Using choices participants made, a hierarchical bayesian multinomial logistic regression was fit to derive mean importance scores with 95% credible intervals (95% Crs) for each quality measure, representing the overall probability of a quality measure being selected as most important. Importance scores were rescaled proportionally from 0 to 100, with the sum of scores for all quality measures adding up to 100. This enabled interpretation of scores as the relative importance of quality measures.

**RESULTS:**

Participants included 61 bereaved parents (median [range] age, 48 [24–74] years; 55 individuals self-identified as women [90.2%]; 1 American Indian or Alaska Native [1.6%], 1 Asian [1.6%], 2 Black or African American [3.3%], 1 Native Hawaiian or Pacific Islander, and 58 White [91.8%]; 58 not Hispanic or Latinx [95.1%]). Highest-priority quality measures by mean importance score included having a child’s symptoms treated well (9.25 [95% Cr, 9.06–9.45]), feeling that a child’s needs were heard by the health care team (8.39 [95% Cr, 8.05–8.73]), and having a goal-concordant end-of-life experience (7.45 [95% Cr, 6.84–8.05]). Lowest-priority quality measures included avoiding chemotherapy (0.33 [95% Cr, 0.21–0.45]), provision of psychosocial support for parents (1.01 [95% Cr, 0.57–1.45]), and avoiding the intensive care unit (1.09 [95% Cr, 0.74–1.43]). Rank-ordering measures by mean importance revealed that symptom management was 9 times more important to parents than psychosocial support for themselves.

**CONCLUSIONS AND RELEVANCE:**

This study found that bereaved parents prioritized end-of-life quality measures focused on symptom management and goal-concordant care while characterizing quality measures assessing their own psychosocial support and their child’s hospital resource use as substantially less important. These findings suggest that future research should explore innovative strategies to measure care attributes that matter most to families of children with advanced cancer.

## Introduction

The passage of the Affordable Care Act in 2010, followed by the Medicare Access and Children’s Health Insurance Program Reauthorization Act of 2015, heralded incentives for quality measurement as a means to enhance care value.^1,2^ However, a substantive gap in current value-based payment models is that quality of care for children with advanced, incurable cancer remains unmeasured.^3–7^ Consequently, end-of-life care for children with cancer in the US varies greatly in intensity, revealing inequities in care provision.^8–11^

Although a set of quality measures exists for adults with advanced cancer,^12^ we previously found that quality measures for adults did not directly translate to the pediatric context owing to developmental considerations in children, the delicate balance of parent and child dyadic decision-making, and what families fundamentally value about advanced childhood cancer care.^4^ Hence, to optimize care value, there is an imminent need to establish end-of-life care quality measures that attend to the preferences and priorities of children with cancer and their families.^13^

In 2 previous studies,^4,6^ we engaged stakeholders in defining and refining what constitutes high-quality end-of-life care for children with cancer. We thereby derived 28 candidate quality measures and narrowed these subsequently to a set of very important measures. Quality measures in the domains of symptom elicitation and management, meeting patient preferences, optimizing family-clinician communication, and interdisciplinary care team engagement were deemed especially important; measures characterizing hospital resource use were perceived as less important overall.^6^ Findings across these studies underscore the need for patient- and family-reported quality measurement. However, a prime limitation of prior studies, particularly those adapting the Delphi technique, is that we cannot distinguish between quality measures of highest and lowest utility to stakeholders.^14^ It is also cognitively challenging for participants to rank-order more than 7 attributes in a modified Delphi process.^15^ Indeed, across 2 studies using expert opinion approaches to hone measures of end-of-life care quality for children with cancer, several dozen measures were endorsed as important, without a prioritization schema.^6,7^

Given that quality measurement is not yet routine in the care of children with advanced cancer, we sought to advance future research and quality improvement initiatives by investigating which quality measures were of highest priority to implement. Our primary objective was to prioritize among 28 candidate quality measures, involving bereaved parents in a quantitative approach to rank order measures in this set.

## Methods

The Yale Human Research Protection Program (HRPP) deemed this survey study exempt from review and waived written informed consent per 45 CFR §46.104 (d)(2)(ii). Verbal informed consent was obtained at the time of participant enrollment, per HRPP guidelines. We adhered to the Strengthening the Reporting of Observational Studies in Epidemiology (STROBE) reporting guideline for cross-sectional studies.

### Study Design and Population

We conducted a cross-sectional discrete choice experiment (DCE) with maximum difference, or best-worst, scaling. Originally designed to estimate consumer preferences in marketing research, this choice-based approach enables better quantitative understanding of the relative importance of each quality measure presented.^16,17^ We recruited parents who had lost a child to cancer and whose children received health care in the US. All participants had spoken and written command of English.

### Participant Recruitment and Enrollment

A volunteer sample of bereaved parents was recruited through social media, outreach to community-based organizations, and snowball sampling. Paid advertisements for the study were posted for 3 months on social media sites (Facebook and Twitter). Concise messages about the study were additionally posted on these sites and Instagram. Using Facebook’s direct messaging platform, we contacted 16 administrators of private groups supporting bereaved parents. Subsequently, 3 private groups agreed to publicize the study to their members.^18,19^ Embracing principles of community-based participatory research, we forged connections with various organizations through the engagement of a community stakeholder and research team member (R.K.); 4 directors of community organizations publicized the study on their respective social media sites and listservs.

Parents expressing interest in participating were requested to complete an online eligibility questionnaire in which we pursued several strategies to minimize inauthentic inquiries: requiring ReCAPTCHA (a service that protects websites from spam and abuse) verification, eliciting US contact information, asking 4 knowledge-based questions, and tracking duplicate responses from the same internet protocol (IP) address via the host site. A study team member (S.D.O. or S.P.) called eligible participants directly to obtain verbal consent. Enrolled participants were then asked to refer other bereaved parents. We confirmed eligibility of 76 parents, 69 of whom were reached by phone, consented, and enrolled; 61 parents completed the DCE, for a participation rate of 88.4%. There were 8 parents who enrolled in the study but did not participate; these individuals were comfortable with spoken and written English and resided in the US.

### Sample Size Considerations

While ideal sample size in a DCE is not well-defined, efficient study design allows for convergence on stable importance scores with relatively small sample sizes.^20^ Calculations of sample size conducted for prior studies^14,21^ indicated that highest-rated items could be differentiated from lowest-rated items with 30 participants. We therefore aimed to recruit at least 30 parents for this study.

### Discrete Choice Experiment Questionnaire

We constructed an electronic DCE questionnaire through Lighthouse Studio version 9 (Sawtooth Software, Inc).^15^ The stem of every question in the DCE was “When thinking about the last weeks of your child’s life, what was most important to you and your family, and conversely, what was least important?” In each of 21 questions in the DCE, participants were presented with a set of 4 quality measures from which they would select the single most and least important measures. Quality measures appearing in the DCE were derived from our prior work and spanned 5 domains: hospital resource use, symptom management, interdisciplinary care, meeting patient and family preferences, and communication.^4,6^ Using a near-balanced incomplete block design, we presented each of 28 quality measures an equal number of times and equally as often with other measures, ensuring level balance and orthogonality. Lighthouse Studio generated numerous versions of the questionnaire such that each participant received different permutations of quality measures across questions in the DCE.^15,22^ At the conclusion of the DCE, participants were asked to self-report standard demographics, including race and ethnicity, and the degree of distress they experienced while answering questions.

### Study Procedures

We administered the DCE questionnaire from January to June 2021. Eligible participants received a unique hyperlink via email. We sent 2 email reminders at 2-week intervals to those who had not yet completed the questionnaire after the initial invitation. Questionnaire access was maintained for 6 weeks. As a token of appreciation, each participant received a $25 gift card.

### Statistical Analysis

We assessed characteristics of the cohort using frequency statistics and measures of central tendency. Parent characteristics included age, gender, race (American Indian or Alaska Native, Asian, Black or African American, Native Hawaiian or Pacific Islander, and White), ethnicity (Hispanic or Latinx or not Hispanic or Latinx), educational attainment, and region of US residence. Child characteristics included age at death, cancer diagnosis, and location of death.

Raw results from the DCE reflect each participant’s selection of the most and least important quality measures from each permutation set presented across items in the questionnaire. We used these data in Lighthouse Studio to fit a multinomial logistic regression model that estimated the probability that a quality measure would be selected as most or least important among a set of measures, relying on the premise that each quality measure was presented in the context of all other measures in a near-balanced design. The model, implemented in a hierarchical bayesian approach, output probabilities for the entire sample and estimated probabilities for individual participants for each quality measure, with individual values shrunken toward entire sample values. Probabilities were rescaled proportionally from 0 to 100, yielding importance scores with the characteristic that the sum of importance scores for all quality measures equaled 100. This proportional rescaling allowed us to reasonably interpret, for example, that a quality measure with an importance score of 10 was perceived to be twice as important as a measure with an importance score of 5.^15,22,23^ For the entire sample, the mean importance score for each quality measure was calculated, along with 95% credible intervals (Crs). Quality measures were rank ordered from highest to lowest mean importance score. At the individual level, we assessed variability in importance score ratings by computing median values and IQRs. Box and whisker plots were created to depict this variation. Analyses were conducted using Lighthouse Studio and R statistical software version 4.0.2 (R Project for Statistical Computing).

## Results

The study exceeded original enrollment goals and included a total of 61 bereaved parents (median [range] age, 48 [24–74] years; 55 individuals self-identified as women [90.2%]; 1 American Indian or Alaska Native [1.6%], 1 Asian [1.6%], 2 Black or African American [3.3%], 1 Native Hawaiian or Pacific Islander, and 58 White [91.8%]; 58 not Hispanic or Latinx [95.1%]). Children who died were predominantly diagnosed with a brain tumor (28 children [45.9%]) or other solid tumor (25 children [41.0%]); 39 children (63.9%) died at home (Table 1). Among 8 parents who enrolled but did not participate, 7 parents (87.5%) had children with brain or other solid tumors.

Most participants (52 parents [85.2%]) reported feeling comfortable or very comfortable answering questions in the DCE. Nearly three-quarters of participants (45 parents [73.8%]) reported little or no distress from the DCE, and no participants reported experiencing a great deal of distress. Many participants (55 parents [90.2%]) reported that participation in this study provided a little, some, or a great deal of benefit to them.

Quality measures receiving the highest mean importance scores included having a child’s symptoms treated well (symptom management domain; 9.25 [95% Cr, 9.06–9.45]), feeling that a child’s needs were heard by the health care team (communication domain; 8.39 [95% Cr, 8.05–8.73]), and having an end-of-life care experience that matched a family’s goals and preferences (meeting patient and family preferences domain; 7.45 [95% Cr, 6.84–8.05]). Quality measures with the lowest mean importance scores included avoiding chemotherapy (hospital resource use domain; 0.33 [95% Cr, 0.21–0.45]), provision of psychosocial support for parents (interdisciplinary care domain; 1.01 [95% Cr, 0.57–1.45]), and avoiding the intensive care unit (hospital resource use domain; 1.09 [95% Cr, 0.74–1.43]). Measures in the domain of hospital resource use were ranked lower in importance overall. Table 2 presents the wording of quality measures as they appeared in the DCE, along with entire sample importance scores for each quality measure.

Measures with the highest and lowest importance scores displayed the least variability across respondents, as assessed by the length of the IQR. Highly scored quality measures with low variability, as shown by the IQR of the importance score, included having a child’s symptoms treated well (0.95) and feeling that a child’s needs were heard (1.41). Low-rated quality measures with low variability, as shown by the IQR of the importance score, included avoiding chemotherapy (0.41), psychosocial support for parents (0.83), and avoiding the intensive care unit (1.32). Midrated quality measures, however, had wider variability, as shown by the IQR of the importance score, with greatest variation in importance scores for care team continuity (5.01) and access to a visiting nurse at home (4.86). These measures pertained to the domain of interdisciplinary care (Table 2; Figure; eFigure in Supplement 1).

## Discussion

To our knowledge, this survey study was the first DCE to engage bereaved parents of children with cancer from across the US. We found that parents prioritized end-of-life care quality measures focused on symptom relief, feeling that a child’s needs were heard, and having a goal-concordant end-of-life experience. Measures limiting use of hospital-based interventions, such as the intensive care unit, cardiopulmonary resuscitation, or chemotherapy, were perceived to be substantially less important. Provision of psychosocial support to parents was among the least important quality measures; satisfactory symptom management was rated as approximately 9 times more important to parents than psychosocial support for themselves.

Parents rated symptom relief as the highest priority for their children near the end of life. Prior pediatric studies^6,14^ similarly found that symptom relief was of great importance to stakeholders, reflecting the burden of multiple symptoms and symptom-related suffering experienced by children with advanced cancer.^24,25^ Simultaneously, we found that psychological support for parents was deprioritized. These findings echo those of several studies exploring good-parent beliefs among caregivers of children with serious illness. Studies have found that parents frequently deferred their own needs in favor of focusing on the needs of their child, remaining at their child’s side, and ensuring that their child felt loved.^21,26^ It is critical to view this relative prioritization in context because the results do not imply that psychological support was unimportant to parents. On the contrary, families facing advanced childhood cancer have previously identified that they valued integration of psychosocial care into overall cancer care.^27^ Parent psychological distress was found to be pervasive and may have exacerbated suffering among children.^28^ Moreover, children and families experienced a range of lasting psychological sequelae.^27,29–31^ Given these findings, consensus standards in pediatric oncology recommend longitudinal involvement of psychosocial clinicians throughout the care of children with cancer.^27,29,32,33^ Nevertheless, substantial barriers exist to receipt of psychosocial care by parents, including reluctance to leave their child’s bedside, difficulty in accessing psychotherapy, time or transportation constraints, and a low number of evidence-based interventions.^33,34^ Commonly held beliefs, barriers, and underlying heuristics may be factors associated with the prioritization schema we observed.^35^

The 2 top-rated quality measures prioritized in this study, symptom management and feeling that a child’s needs were heard, map directly onto measures recently endorsed by the National Quality Forum for ambulatory palliative care in adults. As part of the Palliative Care Quality Measures Project, the American Academy of Hospice and Palliative Medicine, RAND Corporation, and National Coalition for Hospice and Palliative Care jointly developed and tested quality measures pertaining to patient experience.^36^ The 2 patient-reported measures center on feeling heard and understood and receiving sufficient help for pain. These measures have yet to be adapted for use in pediatrics. Although patient experience measures are imperfect, implementation of validated patient experience instruments may be associated with improved clinical outcomes and equity and potential reductions in unnecessary health care use.^37,38^ Given the high priority attributed to patient experience measures in our study and a national call to enhance patient-centeredness of care,^39,40^ next steps in our work include developing a robust instrument to enable children and parents to report on their care experiences in prioritized domains.

Quality measures pertaining to goal concordance, including having an end-of-life care experience that matched a family’s goals and preferences and having one’s child die in a place of the family’s choosing, were among the top 5 most important measures in our study. The priority placed on goal concordance corresponds to widely accepted notions of preference-sensitive end-of-life care.^41,42^ However, operationalizing and concretely measuring goal-concordant care requires researchers to overcome several challenges. Measurement of goal concordance typically requires elicitation of goals and preferences followed by documentation in an electronic health record. Unfortunately, documentation across health systems is neither systematic nor standardized, and interoperability is lacking across different electronic health record systems.^42,43^ Often, documented preferences were found to be nonspecific, rendering it difficult to ascertain post hoc whether decisions were consistent with patient or family preferences. Goals may also shift over time.^41,42,44^ Novel tools that can be used to measure goal concordance may include prospective patient and family experience questionnaires, retrospective questionnaires engaging bereaved caregivers, and artificial intelligence–based methods to capture content in the electronic health record.^41,43,44^ These tools have not yet been implemented in childhood cancer care.

Measurement of hospital resource use ranked low in priority for parents in our study, even though such measures are commonly used to assess population-level end-of-life care quality for individuals with cancer.^3,10,12,45,46^ Other studies similarly found that families expressed ambivalence on measures of hospital use.^4,6^ There may also be unintended consequences of predicating quality on hospital use measures given that many factors, including systemic racism, social determinants of health, and financial incentives for health systems to adhere to publicly reported measures, greatly impact the dynamics of end-of-life care.^47,48^ Taken together, these studies prompt clinicians to reconceptualize high-quality end-of-life care for children with cancer, with a greater emphasis on person-centered measures.^5^

### Limitations

This study has several limitations, including the relative racial, ethnic, and gender homogeneity of participants despite multipronged outreach to parents across the US. To enhance generalizability in subsequent studies, it is imperative that we explore the experiences of fathers, parents who speak languages other than English, individuals who identify as members of historically marginalized groups, and those who may have limited health literacy.^11,49,50^ Mirroring the characteristics of study participants, 8 parents who enrolled in the study but did not participate were comfortable with spoken and written English and resided in the US; the children of 7 of these parents (87.5%) had brain or other solid tumors. Although we did not collect further reasons for nonparticipation from these parents, findings from a prior study^51^ suggest that prolonged grief and cognitive load of questionnaires may be associated with lower rates of study participation among bereaved caregivers. Another limitation of our study was sample size. Albeit sufficient for conduct of a DCE, the small sample size prevented us from pursuing latent class analyses or other analytic approaches to investigate how preferences varied by subgroup. To ensure high questionnaire completion rates and minimize burden on bereaved parents, we did not collect detailed data on parent and child characteristics or evaluate associations between specific family experiences and parent responses. These are crucial considerations for future studies. Notably, few parents in this study cited distress from participation. Most parents reported that the study offered at least some benefit to them,^52,53^ suggesting that it may be feasible in forthcoming research to explore the association between patient and family experiences and end-of-life care preferences. Additionally, we did not hear from patients directly. Knowing how patients with advanced childhood cancer rank these measures may greatly inform future efforts to measure and improve care quality.

## Conclusions

A central challenge to quality measurement in advanced childhood cancer is measuring what families prioritize and balancing family priorities with evaluation of the care delivered by health care teams, hospitals, and health systems. Faced with finite resources and enormous complexities in cancer care delivery, systematic quality measurement has not been implemented for children with advanced illness. As the eminent nineteenth century physicist Lord Kelvin once stated, “If you cannot measure it, you cannot improve it.”^54^ By eliciting priorities from families directly affected by advanced childhood cancer, this survey study’s findings may help clinicians begin to reframe the dialogue around what constitutes high-quality end-of-life care, implement quality measures, and ultimately improve care for thousands of children with advanced cancer who face incurable illness each year.

## Supplementary Material

Supplementary Table 1 SUPPLEMENT 1.
**eFigure**. Distribution of Importance Score Ratings for Quality Measures, Rank Ordered from Most to Least Important

Supplementary File_Data Sharing Statement SUPPLEMENT 2.
Data Sharing Statement

## Figures and Tables

**Figure.**
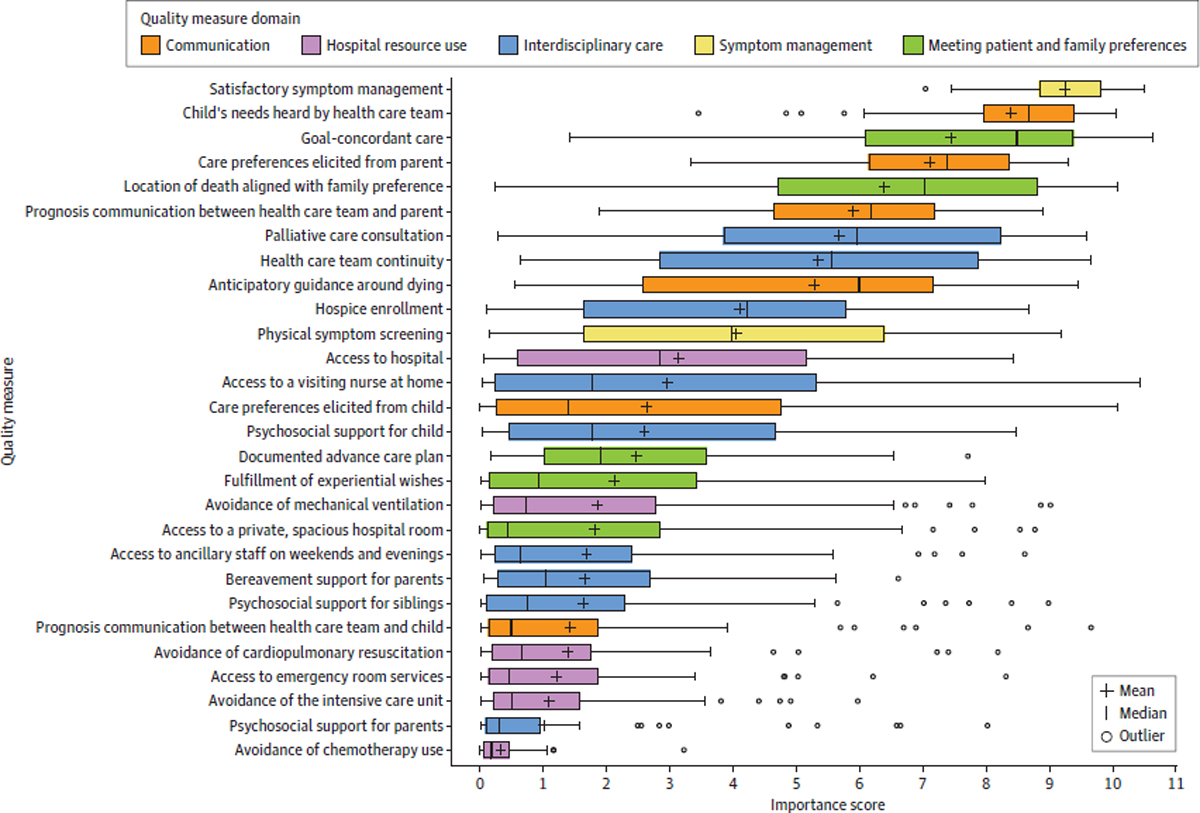
Variation in Importance Score Ratings for Quality Measures Quality measures are rank ordered from most to least important. Boxes indicate IQR for importance scores, with the left edge depicting the 125th percentile and the right edge depicting the 75th percentile; open circles, outliers (defined as importance scores exceeding 1.5 × IQR); vertical lines within boxes, median scores; whiskers, minimum and maximum importance scores; + sign embedded within boxes, mean importance scores for each quality measure across all participants.

**Table 1.** Demographic and Clinical Characteristics of Parent Participants and Their Children

Characteristic	Individuals, No. (%) (N = 61)
Parents	
Age, median (range), y	48 (24–74)
Race	
American Indian orAlaska Native	1 (1.6)
Asian	1 (1.6)
Black or African American	2 (3.3)
Native Hawaiian or Pacific Islander	1 (1.6)
White	56 (91.8)
Ethnicity	
Hispanic or Latinx	3 (4.9)
Not Hispanic or Latinx	58 (95.1)
Gender	
Men	6(9.8)
Women	55 (90.2)
Education	
High school	2 (3.3)
Some college	20 (32.8)
Bachelor's degree	26 (42.6)
Graduate or professional degree	13 (21.3)
Region of US residence	
Northeast	14(23.0)
Midwest	15 (24.6)
South	21 (34.4)
West	11 (18.0)
Children	
Age at death, median (range), y	8 (0–26)
Cancer diagnosis	
Leukemia or lymphoma	8 (13.1)
Solid tumor	25 (41.0)
Brain tumor	28 (45.9)
Location of death	
Home	39 (63.9)
Hospital	21 (34.4)
Hospice	1(1.6)

**Table 2.** Mean and Median Importance Scores for Quality Measures

Quality measure^a^	Domain	Importance score, mean (95% Cr)	Importance score, median (IQR)
Having my child's symptoms treated well	Symptom management	9.25 (9.06–9.45)	9.25 (8.86–9.81)
Feeling that my child's needs were heard by the care team	Communication	8.39 (8.05–8.73)	8.67 (7.97–9.38)
Having an end-of-life care experience that matched our goals and preferences	Meeting patient or family preferences	7.45 (6.84–8.05)	8.48 (6.30–9.38)
Doctors communicating directly with me, a parent/legal guardian, about preferences for care	Communication	7.12 (6.74–7.50)	7.39 (6.31–8.27)
Having my child die in a place of our family's choosing	Meeting patient or family preferences	6.39 (5.70–7.08)	7.02 (4.78–8.78)
Doctors communicating directly with me, a parent/legal guardian, about prognosis	Communication	5.90 (5.45–6.34)	6.17(4.69–7.13)
Receiving support from a palliative care team	Interdisciplinary care	5.68 (5.00–6.36)	5.96 (3.90–8.20)
Having the same oncology team take care of my child throughout the course of treatment	Interdisciplinary care	5.34(4.65–6.03)	5.57 (2.87–7.88)
Receiving guidance about what to expect in the dying process	Communication	5.29 (4.61–5.97)	5.99 (2.68–7.15)
Receiving hospice services	Interdisciplinary care	4.10(3.49–4.71)	4.22 (1.64–5.64)
Being asked regularly about mychild's physical symptoms	Symptom management	4.05 (3.36–4.75)	3.99 (1.64–6.22)
Being able to stay in the hospital for care whenever needed	Hospital resource use	3.14(2.49–3.80)	2.84(0.59–5.16)
Having a visiting nurse help at home	Interdisciplinary care	2.96 (2.20–3.72)	1.77 (0.24–5.10)
Doctors communicating directly with my child about preferences for care	Communication	2.64(1.86–3.41)	1.40 (0.27–4.36)
Psychosocial support for my child	Interdisciplinary care	2.60 (1.97–3.23)	1.78 (0.52–4.64)
Having an advance care plan	Meeting patient or family preferences	2.47 (2.02–2.92)	1.91 (1.09–3.45)
Having experiential wishes (ie, Make-A-Wish, vacations, or trips we wanted to take) fulfilled	Meeting patient or family preferences	2.13 (1.48–2.77)	0.94(0.14–3.28)
Avoiding a ventilator	Hospital resource use	1.86 (1.24–2.47)	0.74(0.22–2.71)
Having a private, spacious hospital room near the end of my child's life	Meeting patient or family preferences	1.82 (1.19–2.44)	0.43 (0.12–2.77)
Having access to ancillary staff on evenings and weekends	Interdisciplinary care	1.69 (1.15–2.22)	0.65 (0.27–2.35)
Receiving support services following my child's death	Interdisciplinary care	1.66 (1.25–2.07)	1.05 (0.29–2.57)
Psychosocial support for other children in my house	Interdisciplinary care	1.63 (1.06–2.20)	0.74(0.10–2.11)
Doctors communicating directly with my child about prognosis	Communication	1.42 (0.87–1.98)	0.50 (0.15–1.85)
Avoiding cardiopulmonary resuscitation	Hospital resource use	1.39 (0.93–1.85)	0.67 (0.21–1.65)
Being able to take mychild to the emergency department	Hospital resource use	1.22 (0.79–1.64)	0.46 (0.15–1.85)
Avoiding the intensive care unit	Hospital resource use	1.09 (0.74–1.43)	0.51 (0.24–1.56)
Psychosocial support for myself	Interdisciplinary care	1.01 (0.57–1.45)	0.30 (0.10–0.93)
Avoiding chemotherapy	Hospital resource use	0.33 (0.21–0.45)	0.17 (0.06–0.47)

Abbreviation: Cr, credible interval.

a Quality measures are worded as they appeared in the administered questionnaire.

## Data Availability

See Supplement 2.

## Key Points

### Question

What do parents who lost a child to cancer prioritize in measuring end-of-life care quality?

### Findings

In this survey study of 61 bereaved parents, respondents prioritized end-of-life quality measures focused on symptom management and goal-concordant care, characterizing quality measures assessing their own psychosocial support and their child’s hospital resource use as substantially less important.

### Meaning

These findings may help set a patient-centered agenda for quality measurement and improvement among children with advanced, incurable cancer.
