# Supplementary material for: Parent Priorities in End-of-Life Care for Children With Cancer
Source: JAMA Netw Open. Author manuscript; Available in PMC 2024 Feb 20. (PMC10878399; doi:10.1001/jamanetworkopen.2023.13503)
Supplement: Supplementary Table 1 — SUPPLEMENT 1. eFigure. Distribution of Importance Score Ratings for Quality Measures, Rank Ordered from Most to Least Important [file NIHMS1965452-supplement-Supplementary_Table_1.pdf]

Supplemental Online Content

Ananth P, Lindsay M, Mun S, et al. Parent priorities in end-of-life care for children with cancer. *JAMA Netw Open.* 2023;6(5):e2313503. doi:10.1001/jamanetworkopen.2023.13503

eFigure. Distribution of Importance Score Ratings for Quality Measures, Rank Ordered from Most to Least Important

This supplemental material has been provided by the authors to give readers additional information about their work.

© 2023 Ananth P et al. *JAMA Network Open.*

eFigure. Distribution of Importance Score Ratings for Quality Measures, Rank Ordered from Most to Least Important

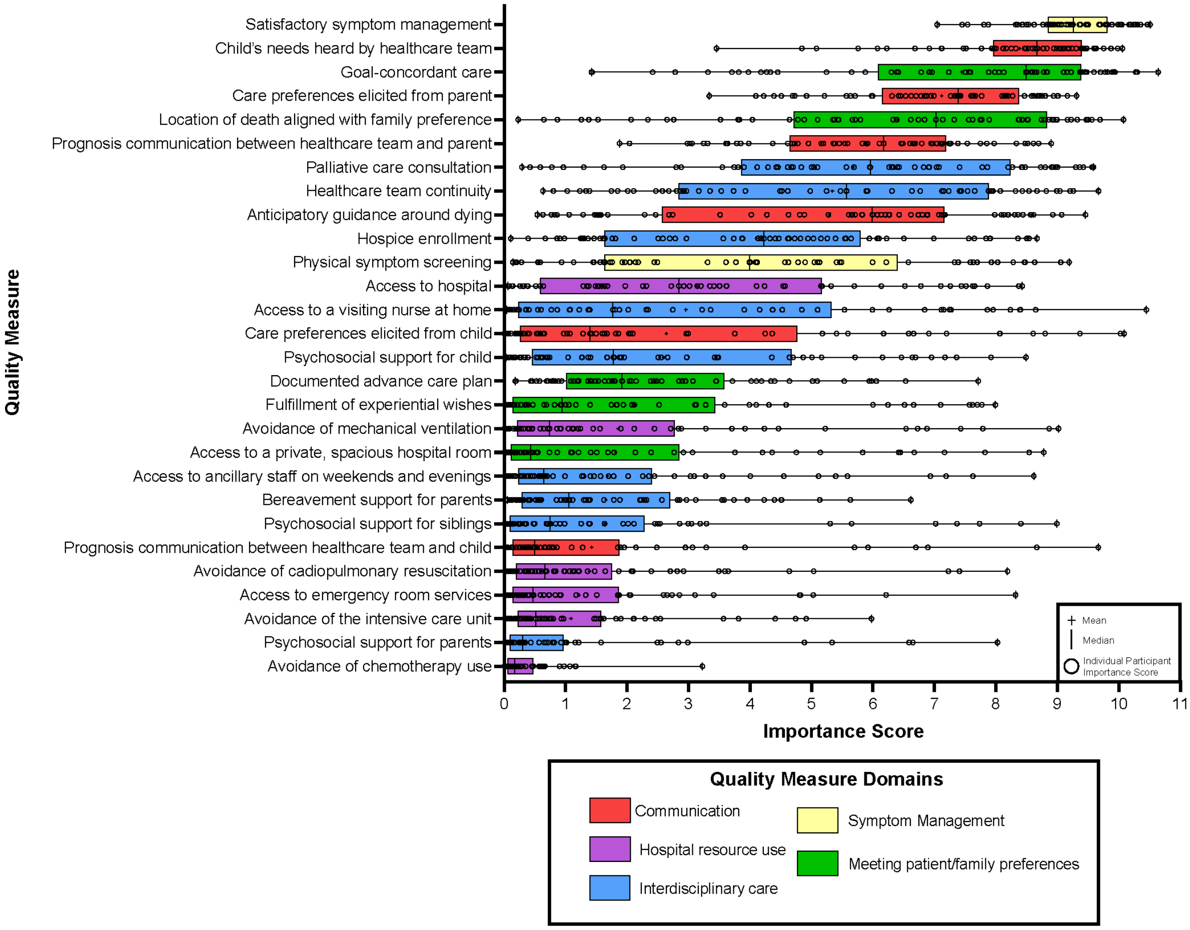

© 2023 Ananth P et al. *JAMA Network Open.*
