# Supplementary material for: Parent Priorities in End-of-Life Care for Children With Cancer
Source: JAMA Netw Open. Author manuscript; Available in PMC 2024 Feb 20. (PMC10878399; doi:10.1001/jamanetworkopen.2023.13503)
Supplement: Supplementary File_Data Sharing Statement — SUPPLEMENT 2. Data Sharing Statement [file NIHMS1965452-supplement-Supplementary_File_Data_Sharing_Statement.pdf]

Data Sharing Statement

Ananth. Parent Priorities in End-of-Life Care for Children With Cancer. *JAMA Netw Open*. Published online May 15, 2023 . doi:10.1001/jamanetworkopen.2023.13503

Data
Data available: Yes
Data types: Deidentified participant data
How to access data: Deidentified data will be made available by the PI (prasanna.ananth@yale.edu) upon reasonable request and with both IRB approval and a signed data use agreement.
When available: With publication

Supporting Documents
Document types: None

Additional Information
Who can access the data: To researchers whose proposed use of the data has been approved
Types of analyses: For any secondary analyses
Mechanisms of data availability: With investigator support, upon both IRB approval of a proposal to conduct secondary data analyses. A signed data use agreement with the PI (Prasanna Ananth) must also be completed.

© 2023 Ananth P et al. *JAMA Network Open*.
